# Variation in the Caprine Keratin-Associated Protein 27-1 Gene is Associated with Cashmere Fiber Diameter

**DOI:** 10.3390/genes11080934

**Published:** 2020-08-13

**Authors:** Mengli Zhao, Huitong Zhou, Yuzhu Luo, Jiqing Wang, Jiang Hu, Xiu Liu, Shaobin Li, Zhiyun Hao, Xiayang Jin, Yize Song, Xinmiao Wu, Liyan Hu, Jon G. H. Hickford

**Affiliations:** 1 Gansu Key Laboratory of Herbivorous Animal Biotechnology, College of Animal Science and Technology, Gansu Agricultural University, Lanzhou 730070, China; 18394187234@163.com (M.Z.); luoyz@gsau.edu.cn (Y.L.); huj@gsau.edu.cn (J.H.); liuxiu@gsau.edu.cn (X.L.); lisb@gsau.edu.cn (S.L.); haozy2018@163.com (Z.H.); jinxy@st.gsau.edu.cn (X.J.); songyz@st.gsau.edu.cn (Y.S.); wuxinmiao2020@163.com (X.W.); huliyan2020@163.com (L.H.); 2 International Wool Research Institute, College of Animal Science and Technology, Gansu Agricultural University, Lanzhou 730070, China; huitong.zhou@lincoln.ac.nz; 3 Gene-Marker Laboratory, Faculty of Agriculture and Life Sciences, Lincoln University, Lincoln 7647, New Zealand

**Keywords:** KAP27-1 gene (*KRTAP27-1*), mean fiber diameter, skin, Longdong cashmere goats

## Abstract

Variation in some caprine keratin-associated protein (KAP) genes has been associated with cashmere fiber traits, but many KAP genes remain unidentified in goats. In this study, we confirm the identification of a KAP27-1 gene (*KRTAP27-1*) and describe its effect on cashmere traits in 248 Longdong cashmere goats. A polymerase chain reaction–single strand conformation polymorphism (PCR-SSCP) analysis was used to screen for sequence variation in this gene, and three sequence variants (named *A* to *C*) were found. These sequences have the highest similarity (77% identity) to a human *KRTAP27-1* sequence, while sharing some homology with a predicted caprine *KRTAP27-1* sequence ENSCHIG00000023347 in the goat genome construct (ARS1:CM004562.1) at chromosome 1 position 3,966,193–3,973,677 in the forward strand. There were two single nucleotide polymorphisms (SNPs) detected in the coding sequence, including one nonsynonymous SNP (c.413C/T; p.Ala138Val) and one synonymous SNP (c.495C/T). The *C* variant differed from *A* and *B* at c.413C/T, having cytosine in its nucleotide sequence, while the *B* variant differed from *A* and *C* at c.495C/T, having thymine in its nucleotide sequence. Goats of the genotypes *AB* and *BB* produced cashmere fibers of higher mean fiber diameter (MFD) than goats of genotype *AA*, but no difference in MFD was detected between the *AB* and *BB* goats. These results suggest that *B* is associated with increased MFD. Expression of the caprine *KRTAP27-1* sequence was predominantly detected in the skin tissue of goats but not or only weakly detected in other tissues, including longissimus dorsi muscle, heart, kidney, liver, lung and spleen.

## 1. Introduction

Cashmere fiber is produced by secondary hair follicles. The fiber has the characteristics of being light, soft, strong, elastic and a good thermal insulator, and it is accordingly referred to as ‘soft gold’, as it plays an important role in the textile industries [1]. The price paid for cashmere fibers is typically higher than that for mohair or wool. With cashmere fiber yield, curly fiber length and mean fiber diameter (MFD) are the most important traits in determining quality.

Keratin-associated proteins (KAPs) and keratins are the major components of cashmere fibers. The KAPs form a matrix and cross-link with cysteine residues on the keratins and other KAPs, through the formation of disulfide bonds. They typically possess a high content of either cysteine or glycine and tyrosine, and they have historically been classified into three broad groups based on their amino acid composition: the high sulfur (HS) group with less than 30 mol% cysteine, the ultrahigh sulfur (UHS) group with more than 30 mol% cysteine and the high glycine/tyrosine (HGT) group containing 35 to 60 mol% glycine and tyrosine [2].

To date, over 100 KAP genes (*KRTAPs*) from 29 families have been identified in mammalian species [2,3,4], but only 15 *KRTAPs* from 11 families have been reported in goats [5,6,7]. Among these caprine *KRTAPs*, nucleotide sequence variation in six has been reported to be associated with some cashmere fiber traits, including variation in *KRTAP8-2* [8], *KRTAP15-1* [5], *KRTAP20-1* [9], *KRTAP20-2* [10], *KRTAP24-1* [6] and *KRTAP28-1* [7].

Based on the similarity of the *KRTAP* sequences among species, some *KRTAPs* have been identified in sheep and goats using the human *KRTAP* sequences as reference points, including ovine *KRTAP26-1* [11], ovine *KRTAP22-1* [12], caprine *KRTAP20-1* [9] and caprine *KRTAP20-2* [10].

The KAP27 protein is from a HS-KAP family that currently contains a single family member. To date, the encoding gene called *KRTAP27-1* has been described in humans ([13]; GenBank sequence AB096937), and an Ensembl-predicted caprine *KRTAP27-1* sequence ENSCHIG00000023347 has been located in the goat genome construct (ARS1: CM004562.1) at chromosome 1 position 3,966,193–3,973,677 in the forward strand. While this sequence shares homology with the human *KRTAP27-1* sequence [13], the gene model predicted in the construct contains two introns, with this not having been observed in any of the other caprine or ovine *KRTAP* sequences reported to date, including caprine *KRTAP8-2* [8], *KRTAP15-1* [5], *KRTAP20-1* [9], *KRTAP20-2* [10], *KRTAP24-1* [6] and *KRTAP28-1* [7] or ovine *KRTAP26-1* [11] and *KRTAP22-1* [12]. In contrast GenBank describes a gene (LOC102181992) located at positions 3,968,607–3,969,209 in sequence NC_030808.1 (ASM170441v1) with an uninterrupted coding sequence of 603 nucleotides and which has 99% homology to a predicted sheep *KRTAP27-1*-like sequence (LOC114109388) and a predicted sheep *KRTAP27-1* sequence (LOC101106296).

In this study, we report the identification of intronless *KRTAP27-1* sequences amplified directly from goat DNA and describe variation in these sequences and the locations of expression of this gene; we then describe the association of this gene with cashmere fiber traits in Longdong cashmere goats.

## 2. Materials and Methods 

### 2.1. Cashmere Goats and Sample Collection

Two hundred forty-eight Longdong cashmere goats reared at the Yusheng Cashmere Goat Breeding Company in Huan County of the Gansu Province of China were investigated. The 248 Longdong cashmere goats were the progeny of eight unrelated sires. These goats included 196 females and 52 males. At twelve months of age (spring time), cashmere fibers were collected from individual goats (live weight 21.7 ± 0.92 kg) that were in the anagen phase of fiber growth by combing, and the raw weights of the collected secondary or cashmere fibers from individual goats were measured. A fiber sample from the mid-side region of each goat was taken for testing MFD and curly fiber length. The MFD and curly fiber length of each goat were measured using the Optical Fiber Length and Diameter Analyzer OFDA4000 (EPCO, Shanghai, China).

Blood samples from these goats were collected on Munktell TFN paper (Munktell Filter AB, Falun, Sweden). Genomic DNA for polymerase chain reaction (PCR) amplification was purified from 1.2-mm punches of the dry blood spots, following the procedure described by Zhou et al. [14].

Three 3-year-old female Longdong cashmere goats were slaughtered to collect tissue samples for reverse-transcription PCR (RT-PCR) analysis, including skin, longissimus dorsi muscle, heart, kidneys, liver, lung and spleen tissue samples. These samples were immediately frozen in liquid nitrogen upon collection.

The collection of caprine blood and skin samples were approved by Gansu Agricultural University, and the feeding of these goats was in accordance with the guidelines for care of experimental animals established by the Ministry of Science and Technology of the People’s Republic of China (Approval number 2006-398).

### 2.2. Polymerase Chain Reaction–Single Strand Conformation Polymorphism (SSCP) Analysis of Caprine KRTAP27-1 

The coding sequence of human *KRTAP27-1* (GenBank sequence AB096937) was used to BLAST search the Caprine Genome Assembly ASM170441v1. The sequence that shared the greatest similarity with the human *KRTAP27-1* sequence was assumed to be the caprine *KRTAP27-1* sequence. Based on this caprine sequence, two PCR primers (5′-ACTCAATCAAGGATTTTCAC-3′ and 5′-GCAGATTCTTCACTCAGTAGG-3′) were designed to amplify the entire coding region of the putative caprine *KRTAP27-1*. These primers were synthesized by the Sangon Biotechnology Company Limited (Shanghai, China).

Amplifications were performed in a 20-µL reaction containing the genomic DNA purified from a 1.2-mm punch of dried blood, 0.25 µM of each primer, 1 × PCR buffer (supplied with DNA polymerase enzyme), 150 µM of each deoxyribonucleoside triphosphate (dNTP) (Takara, Dalian, China), 2.5 mM Mg^2+^ and 0.5 U of *Taq* DNA polymerase (Takara, Dalian, China). The PCR amplification profile was comprised of an initial denaturation at 94 °C for 5 min, followed by 35 cycles of 94 °C for 30 s, 60 °C for 30 s and 72 °C for 30 s, and then a final extension for 5 min at 72 °C. The thermal cycling was performed in Bio-Rad S1000 thermal cyclers (Bio-Rad, Hercules, CA, USA), and the presence of PCR products was confirmed and qualitatively assessed using 1.0% agarose gel electrophoresis in 1 × TBE buffer.

Individual samples containing a 1.0-µL aliquot of a single PCR amplicon and 7.5 µL of loading dye (98% formamide, 10 mM ethylenediaminetetraacetic acid (EDTA), 0.025% bromophenol blue and 0.025% xylene cyanol) were denatured at 95 °C for 10 min, then quickly cooled on wet ice and finally loaded onto 16 cm × 18 cm, 10% acrylamide:bisacrylamide (37.5:1) (Bio-Rad, Hercules, CA, USA) gel. Electrophoresis was undertaken in 0.5 × TBE buffer at 210 V and 16.5 °C for 20 h using Protean II xi cells (Bio-Rad, Hercules, CA, USA). The gels were silver-stained using the method of Byun et al. [15].

### 2.3. Sequencing of KRTAP27-1 Variants and Sequence Analyses

After PCR-SSCP analysis, amplicons from goats that appeared to be homozygous based on the observed PCR-SSCP banding patterns were sequenced directly, and in both directions, at the Beijing Genomics Institute (Beijing, China). Variants that were only found in a heterozygote form were sequenced using the method described by Gong et al. [16].

Sequence alignments, the DNA sequence translations and phylogenetic tree construction were undertaken using DNAMAN version 5.2.10 (Lynnon BioSoft, Vaudreuil, QC, Canada).

### 2.4. Reverse-Transcription Polymerase Chain Reaction (RT-PCR) Analysis

Total RNA from the tissue samples was extracted using Trizol reagent (Invitrogen, Carlsbad, CA, USA). The quality and concentration of RNA were ascertained using 2% agarose gel electrophoresis and UV spectrophotometry, respectively. Complementary DNA (cDNA) was synthesized using the PrimeScript RT Reagent Kit with gDNA Eraser (Perfect Real Time, Takara, Dalian, China) according to the manufacturer’s instructions and then amplified using a set of inner primers (5′-TCGGCAGTGTTGGAGTGTGT-3′ and 5′-GGACAAGACTTCGCCATGTTGC-3′) that were designed to amplify a smaller portion (110 bp) of the putative caprine *KRTAP27-1*. The amplification conditions and thermal profile were the same as for the genomic amplifications described above, but the genomic DNA template was replaced with a 0.8-µL aliquot of the cDNA. The RT-PCR products were visualized using 1.0% agarose gel electrophoresis. The caprine β-actin gene (*ACTB*) was used as a positive control reference gene and amplified using the primers 5′-AGCCTTCCTTCCTGGGCATGGA-3′ and 5′-GGACAGCACCGTGTTGGCGTAGA-3′.

### 2.5. Statistical Analyses

All data were analyzed using IBM SPSS Statistics version 24.0 (IBM, NY, USA) software. Pearson correlation coefficients between MFD, cashmere yield and curly fiber length were determined. 

The effect of variation in *KRTAP27-1* on the three cashmere traits was assessed using general linear mixed-effect models (GLMMs), but only those genotypes that occurred at a frequency of over 5% were analyzed. Sire (*n* = 8) and gender were found to affect all the fiber traits (*p* < 0.05), and they were therefore included in the GLMMs as a random factor and a fixed factor, respectively. Birth rank was not found to affect these traits, and thus it was not included in the models. Only main effects were tested. A Bonferroni correction was applied to reduce the probability of false positive results during the multiple comparisons in these models. Unless indicated, all *p* values were considered statistically significant when *p* < 0.05.

## 3. Results

### 3.1. Identification and Chromosomal Location of Caprine KRTAP27-1

**Figure 1:**
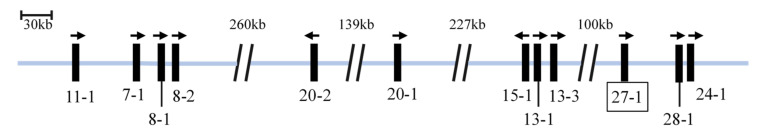
*KRTAPs* identified on goat chromosome 1. Eleven identified *KRTAPs* and the newly identified *KRTAP27-1* (boxed) are located on goat chromosome 1. Vertical bars and arrows represent *KRTAPs* and the direction of transcription, respectively, with the names of the genes being indicated below (i.e., 11-1 represents *KRTAP11-1*). The distances between these genes are approximate and refer to the caprine chromosome 1 sequence (NC_030808.1).

A BLAST search of the Caprine Genome Assembly ASM170441vl using the human *KRTAP27-1* sequence (GenBank Accession Number AB096937) revealed that the most homologous region (with 77% identity) was on caprine chromosome 1. This region contained a 603-bp open reading frame (ORF) (NC_030808.1: nt 3,968,607 to 3969209) that would encode a polypeptide with the most common amino acid being serine (17.5 mol%), followed by cysteine (9.5 mol%). Eleven previously described caprine KAP genes were also found in this region, including caprine *KRTAP11-1, KRTAP7-1, KRTAP8-1, KRTAP8-2, KRTAP20-2, KRTAP20-1, KRTAP15-1, KRTAP13-1*, *KRTAP13-3*, *KRTAP28-1* and *KRTAP24-1* (Figure 1).

### 3.2. Sequence Variation in Caprine KRTAP27-1

**Figure 2:**
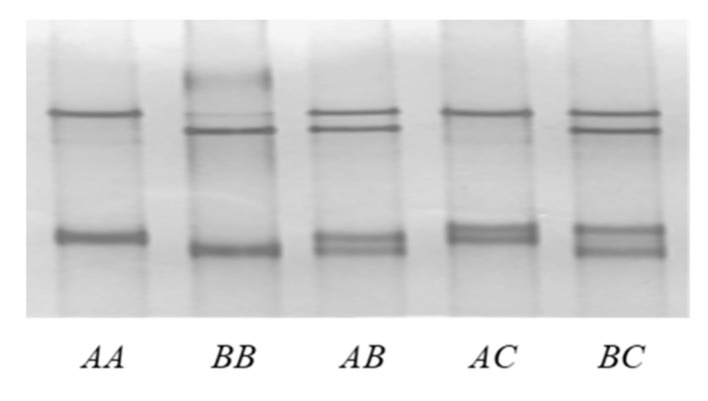
Polymerase chain reaction–single-strand conformation polymorphism (PCR-SSCP) analysis of caprine *KRTAP27-1.* Three different banding patterns, representing three different variants (*A*–*C*), are shown in either homozygous or heterozygous forms.

Three different PCR-SSCP banding patterns representing three variants (named *A, B* and *C*) were identified in the Longdong cashmere goats investigated (Figure 2).

**Figure 3:**
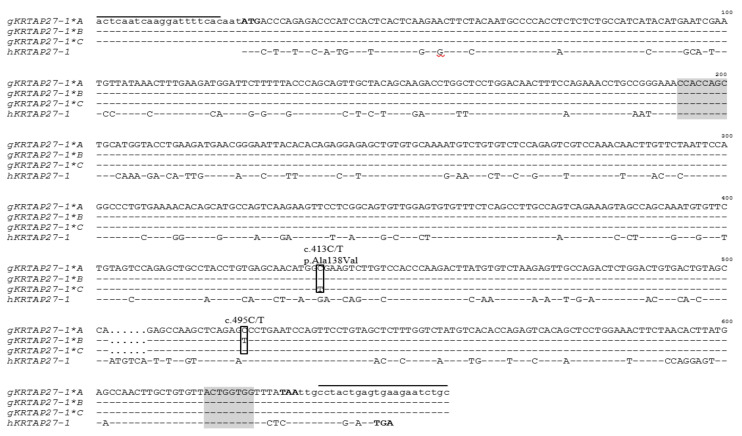
Alignment of the *KRTAP27-1* variants identified in goats with the human *KRTAP27-1* coding sequence. The prefixes “g” and “h” stand for goat and human sequences, respectively, and homology with caprine KRTAP27-1*A is indicated by dashes. Nucleotides in the coding region are shown in uppercase, while those in the noncoding region are in lowercase. The primer-binding regions are indicated with horizontal lines above the sequences, and the start codon (ATG) and stop codon (TAA and TGA) are shown in bold. Two single nucleotide polymorphisms (SNPs) identified in caprine *KRTAP27-1* are indicated. A reverse complementary *Chi* sequence (CCACCAGC) and a *Chi*-like sequence (ACTGGTGG) are shaded. The numbering of the nucleotides and amino acids follows the Human Genome Variation Society (HGVS) nomenclature (http://varnomen.hgvs.org/). The GenBank Accession Numbers of these sequences are MN934937 (gKRTAP27-1*A), MN934938 (gKRTAP27-1*B), MN934939 (gKRTAP27-1*C) and AB096937 (*hKRTAP27-1*). The predicted caprine *KRTAP27-1* sequence described in Caprine Genome Assembly sequence NC_030808.1 is identical to gKRTAP27-1*A and hence is not shown separately.

Sequencing of PCR amplicons representing the three different banding patterns revealed three unique nucleotide sequences (*A, B* and *C*), with sequence *A* being identical to the Caprine Genome Assembly (NC_030808.1). Two single nucleotide polymorphisms (SNPs) were found, with one (c.495C/T) being synonymous and the other (c.413C/T) being nonsynonymous, that would lead to a putative amino acid change of p.Ala138Val (Figure 3). A reverse complementary *Chi* sequence and a *Chi*-like sequence were detected in these variants. These variant sequences were deposited into GenBank and allocated the Accession Numbers MN934937, MN934938 and MN934939. In the 248 Longdong cashmere goats investigated, variants *A*, *B* and *C* were found at frequencies of 71.37%, 23.79% and 4.84%, respectively. Five genotypes (*AA*, *BB*, *AB*, *AC* and *BC*) were found, with frequencies of 52.02%, 5.65%, 32.66%, 6.05% and 3.63%, respectively.

### 3.3. Phylogenetic Analysis of Caprine KAP27-1

**Figure 4:**
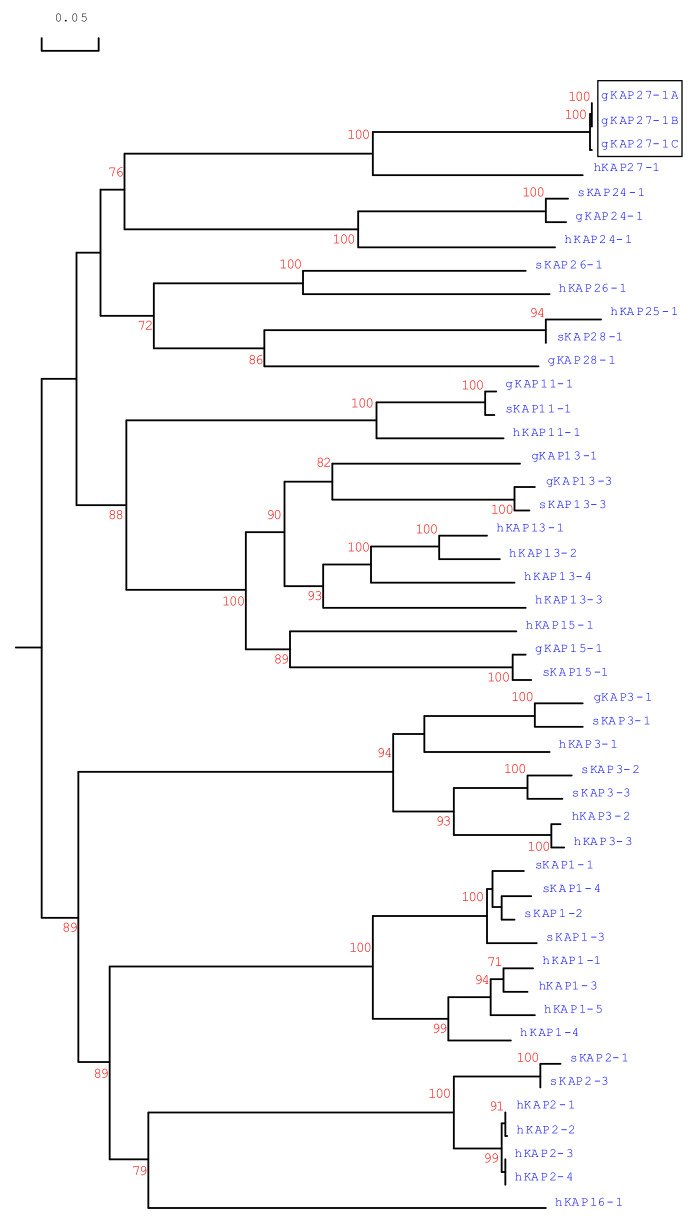
Phylogenetic tree of HS-KAPs from goats, sheep and humans. The amino acid sequences of HS-KAPs from humans, sheep and goats were used to construct the tree. The sequences from goats are represented with a prefix “g”, while the sequences from humans and sheep are indicated with the prefixes “h” and “s”, respectively. The putative newly identified caprine KAP27-1 sequences are shown in a box. The numbers at the forks represented the bootstrap confidence values and only those equal to or higher than 60% are shown. The sequence of gKAP28-1 was sourced from Wang et al. [7], and the other KAP sequences were retrieved from GenBank with Accession Numbers: NM_001285774 (gKAP3-1), NM_001285767.1 (gKAP11-1), AY510115 (gKAP13-1), JX426138 (gKAP13-3), NM_001285771 (gKAP15-1), MG996011 (gKAP24-1), X01610 (sKAP1-1 and sKAP1-4), HQ897975 (sKAP1-2), NM_001159761 (sKAP1-3), P02443 (sKAP2-1), U60024 (sKAP2-3), M21099 (sKAP3-1), M21100 (sKAP3-2), M21103 (sKAP3-3), HQ595352 (sKAP11-1), JN377429 (sKAP13-3), MH742372 (sKAP15-1), JX112014 (sKAP24-1), KX644903 (sKAP26-1), MN053920 (sKAP28-1), NM_030967.2 (hKAP1-1), NM_030966.1 (hKAP1-3), NM_001257305.1 (hKAP1-4), NM_031957.1 (hKAP1-5), NM_001123387.1 (hKAP2-1), NM_033032.2 (hKAP2-2), NM_001165252.1 (hKAP2-3), NM_033184.3 (hKAP2-4), NM_031958.1 (hKAP3-1), NM_031959.2 (hKAP3-2), NM_033185.2 (hKAP3-3), NM_175858.2 (hKAP11-1), NM_181599.2 (hKAP13-1), NM_181621.3 (hKAP13-2), NM_181622.2 (hKAP13-3), NM_181600.1 (hKAP13-4), NM_181623.2 (hKAP15-1), NM_001146182.1 (hKAP16-1), NM_001085455.2 (hKAP24-1), NM_001128598.1 (hKAP25-1), NM_203405.1 (hKAP26-1) and AB096937.1 (hKAP27-1).

A phylogenetic analysis of the caprine *KRTAP27-1* sequences, along with all of the other HS-*KRTAPs* identified in identified in goats, sheep and humans, revealed that the three caprine *KRTAP27-1* sequences identified were most closely related to human *KRTAP27-1*, and less closely related to all of the other HS-*KRTAPs* (Figure 4). This suggests the three sequences identified represent sequence variants of caprine *KRTAP27-1*, and thus they were named CAPHI-KRTAP27-1*A, CAPHI-KRTAP27-1*B and CAPHI-KRTAP27-1*C, based on the nomenclature proposed by Gong et al. [17].

### 3.4. Expression of Caprine KRTAP27-1

**Figure 5:**
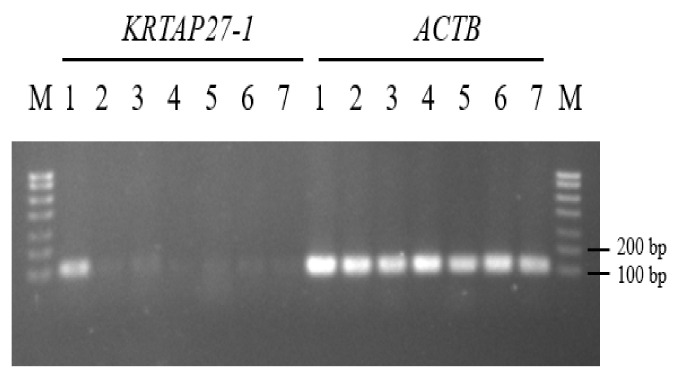
Reverse-transcriptase polymerase chain reaction (RT-PCR) analysis of caprine *KRTAP27-1* and *ACTB* in various tissues of Longdong cashmere goats. These tissues are (1) skin, (2) heart, (3) liver, (4) spleen, (5) lung, (6) kidney and (7) longissimus dorsi muscle. M: DNA marker (Direct-Load PCR Marker II, GenStar, Beijing, China).

The RT-PCR analysis of seven different tissues from the Longdong cashmere goats revealed that caprine *KRTAP27-1* expression was predominantly detected in the skin tissue but was not detected or only weakly detected in the longissimus dorsi muscle, heart, kidney, liver, lung and spleen tissues (Figure 5).

### 3.5. Correlation between Cashmere Fiber Traits

A moderate correlation was found between curly fiber length and cashmere fiber yield (r = 0.496; *p* < 0.001), while MFD had a weak correlation with cashmere fiber yield (r = 0.252; *p* < 0.001) and curly fiber length (r = 0.209; *p* < 0.001).

### 3.6. Association between KRTAP27-1 Genotypes and Cashmere Fiber Traits

Of the three variants found in the Longdong cashmere goats, variant *C* occurred at a frequency of less than 5%, and hence the goats carrying *C* in their genotype were removed from the association study. There was no difference in the number of sires before and after removing the goats (*n* = 24) with genotypes *BC* and *AC*. This left 224 goats of three genotypes (*AA*, *AB* and *BB*), which were used for the association analysis. Goats of genotype *AB* or *BB* were found to produce cashmere fibers with higher MFD (*p* = 0.026) than goats of genotype *AA* (Table 1), suggesting that variant *B* is associated with increased MFD. Variation in *KRTAP27-1* was not found to affect either cashmere fiber yield or curly fiber length.

## 4. Discussion

This study confirms the identity of a caprine KAP gene on goat chromosome 1, which putatively encodes a HS-KAP protein that is most similar to the human KAP27-1 and the predicted caprine *KRTAP27-1* sequences in Ensembl and GenBank. However, the Ensembl-predicted caprine *KRTAP27-1* sequence ENSCHIG00000023347 located in the goat genome construct (ARS1:CM004562.1) at chromosome 1 position 3,966,193–3,973,677 contains two introns, with this not having been observed in the human *KRTAP27-1* sequence or any of the other caprine or ovine *KRTAP* sequences reported to date, including caprine *KRTAP8-2* [8], *KRTAP15-1* [5], *KRTAP20-1* [9], *KRTAP20-2* [10], *KRTAP24-1* [6] and *KRTAP28-1* [7] or ovine *KRTAP26-1* [11] and *KRTAP22-1* [12]. This would suggest the construct may contain assembly errors. Regions that are highly polymorphic and repetitive, such as the *KRTAP* genes, are particularly difficult to assemble [18], and the analysis of ARS1 by Li et al. [19] revealed that some sequences containing essential genes were missing from ARS1, despite their presence in the sequenced ARS1 individuals. This suggests that the long-read sequencing technologies or the assembling methods used to assemble ARS1 still have limitations that warrant further improvements.

In contrast to other HS-KAPs, this protein contains a high level of serine and moderate level of cysteine. Having a higher level of serine than cysteine has been described previously for a number of HS-KAPs, including caprine KAP15-1 [5], caprine KAP24-1 [6], ovine KAP13-3 [20], ovine KAP24-1 [21] and ovine KAP26-1 [11]. The precise role of KAP serine residues in animal fiber is unknown, but these serine residues may possibly be phosphorylated [22], especially as the phosphorylation of keratins can affect keratin assembly and organization [23]. It is therefore possible that serine may play a role in regulating interactions between KAPs and keratins and consequently affect the fiber structure.

The specific expression of caprine *KRTAP27-1* in skin is consistent with the expression pattern of other *KRTAPs* described in goats, such as *KRTAP15-1* [5], *KRTAP20-1* [9] and *KRTAP20-2* [10], but it contrasts with the expression pattern for human *KRTAP27-1* [24], with expression of the gene apparently not found in all of the tissues taken from the Caucasians investigated, including skin and hair follicles. Accordingly, Rogers et al. [24] suggest the possibility of individual- or population-specific expression of this gene.

The detection of two SNPs in the 603-bp *KRTAP27-1* coding sequence amplified gives a density of approximately 3.3 SNPs per Kb. This density is lower than a previously reported average density of 4.9 SNPs per Kb across the entire ovine genome [25], with this ovine density estimate based not only just on coding sequence but also on the more plentiful and arguably less functionally constrained noncoding parts of the genome. This stated, ovine *KRTAPs* do appear to have higher SNP densities that other parts of the sheep genome, with a density of over 20 SNPs/Kb having been reported for ovine *KRTAP1-3* [26] and *KRTAP1-4* [27]. This may be because there is evidence to suggest that gene conversion or nonreciprocal genetic exchange contributes to the accumulation of sequence variation in the *KRTAPs* [5,6,28,29], and these recombination events may be facilitated by the *Chi* (5′-GCTGGTGG-3′) and *Chi-*like sequences within the genes [5,6,28,30]. In this respect, the presence of *Chi* and *Chi*-like sequences in *KRTAP27-1* suggests recombination mechanisms may be creating sequence diversity in this gene as well.

Variants *A* and *B* differed by one synonymous SNP, c.495C/T; hence, the finding that these variants differentially affected MFD is notable. While synonymous SNPs do not lead to amino acid changes, they have been reported to affect the stability and secondary structure of mRNA, and hence they can have functional significance [31,32]. The possibility also exists that the effect detected may be due to other unidentified but functionally important SNPs located in proximity to the amplified region. For example, given that *KRTAPs* are clustered, it is possible that the association with MFD detected for *KRTAP27-1* may be due to linkage with other nearby *KRTAPs*. This is supported by the findings of similar phenotypic effects for *KRTAP15-1* [5], *KRTAP24-1* [6] and *KRTAP28-1* [7], which are physically closed to *KRTAP27-1* on the same chromosome.

The finding that *KRTAP27-1* variation is associated with increased cashmere fiber MFD is noteworthy. This stated, selection for lower MFD may lead to reduced cashmere yields, as these two traits have a weak positive correlation, although the result of the association analysis whereby *KRTAP27-1* variation does not appear to affect yield would suggest it might be possible. Given the high heritabilities reported for cashmere MFD [33,34], it could be concluded that greater progress in breeding for reduced MFD may be achieved through traditional selection approaches.

## Figures and Tables

**Table 1:** Association of caprine *KRTAP27-1* genotypes with cashmere fiber traits in Longdong cashmere goats (Mean ± SE) ^1.^

Fiber Trait	Raw Mean ± SE (*n* = 248)	(Mean ± SE) ^1^			*p* Value
		*AA* (*n* = 129)	*AB* (*n* = 81)	*BB* (*n* = 14)	
Cashmere fiber yield (g)	397.5 ± 3.18	420.4 ± 4.88	417.0 ± 5.75	412.3 ± 11.45	0.721
Mean fiber diameter (μm)	13.3 ± 0.03	**13.4 ± 0.05 ^a^**	**13.5 ± 0.06 ^b^**	**13.7 ± 0.11 ^b^**	**0.026**
Curly fiber length (cm)	4.1 ± 0.03	4.4 ± 0.05	4.3 ± 0.06	4.2 ± 0.12	0.127

^1^ Estimated marginal means and standard errors (SE) of those means from general linear mixed-effects models. Means that do not share a superscript letter (a or b) within rows are significantly different at *p* < 0.05 and are shown in bold.
